# Greener, Faster, Stronger: The Benefits of Deep Eutectic Solvents in Polymer and Materials Science

**DOI:** 10.3390/polym13030447

**Published:** 2021-01-30

**Authors:** Yeasmin Nahar, Stuart C. Thickett

**Affiliations:** School of Natural Sciences—Chemistry, University of Tasmania, Hobart, TAS 7001, Australia; yeasmin.nahar@utas.edu.au

**Keywords:** deep eutectic solvents, green chemistry, sustainability, radical polymerization, hydrogels, polycondensation, molecularly imprinted polymers, electrochemical polymerization, polymer monoliths

## Abstract

Deep eutectic solvents (DESs) represent an emergent class of green designer solvents that find numerous applications in different aspects of chemical synthesis. A particularly appealing aspect of DES systems is their simplicity of preparation, combined with inexpensive, readily available starting materials to yield solvents with appealing properties (negligible volatility, non-flammability and high solvation capacity). In the context of polymer science, DES systems not only offer an appealing route towards replacing hazardous volatile organic solvents (VOCs), but can serve multiple roles including those of solvent, monomer and templating agent—so called “polymerizable eutectics.” In this review, we look at DES systems and polymerizable eutectics and their application in polymer materials synthesis, including various mechanisms of polymer formation, hydrogel design, porous monoliths, and molecularly imprinted polymers. We provide a comparative study of these systems alongside traditional synthetic approaches, highlighting not only the benefit of replacing VOCs from the perspective of environmental sustainability, but also the materials advantage with respect to mechanical and thermal properties of the polymers formed.

## 1. Introduction

In the age of green chemistry and an increased focus on process sustainability, there is an increased awareness within the research community towards the use of traditional organic solvents [1]. In typical chemical processes, solvents account for the bulk of the total reaction mass and associated waste production, which raises potentially significant environmental concerns. As numerous solvents are derived from petroleum-based feedstocks, there are also concerns regarding the long-term security and appropriate use of these chemicals. The combination of these factors has seen chemists seek alternative solvent systems to address these concerns. Examples of such alternative, more environmentally friendly solvents include dihydrolevoglucosenone (Cyrene^TM^) [2], RodiaSolv© PolarClean [3,4] and bio-derived lignin pyrolysis oil methyl ether [5], in particular various substituted anisoles [6].

In addition to the above examples, systems known as “deep eutectic solvents” (DESs) are attracting significant attention in numerous areas of chemistry and materials science as novel alternatives to typical solvents due to their unique properties and the potential benefits their use confers. Additionally, in contrast to aromatic hydrocarbons, long-chain aliphatic hydrocarbons and chlorinated solvents, DES systems have both low purchase cost and low monetary impact values with respect to solvent emissions [7], which is an important consideration for solvent use on an industrial scale. DES systems have been known since the end of the 19th century [8], typically referred to as “eutectic mixtures” but now more frequently as deep eutectic solvents [9] as will be the case in this review, or alternatively low transition temperature mixtures (LTTMs) [10]. The rapid developments in the use of DES systems seen over the past twenty years have emerged due to their appealing properties they possess, in particular their ease of preparation compared to ionic liquids (ILs [11]). DES systems are powerful solvents that exhibit negligible volatility, are non-flammable and certain classes are electrically conductive; they can also be recycled through recovery of the respective solvent components [12,13,14]. DES systems are largely considered to be non-toxic [15], although it is worth noting that DESs consisting of individual components that are considered to be safe show increased cytotoxicity relative to aqueous mixtures of the components [16]. Natural deep eutectic solvents (often referred to as NADES [17,18]) are perhaps even more appealing, as the components are derived from bio-sourced and/or renewable feedstocks. The applications of DES systems are diverse given their unique properties, and have seen application in numerous areas including metal nanoparticle synthesis [19], carbon dioxide fixation [20], modification [21] and solubilization [22] of cellulose, RNA extraction [23], preparation and modification of polymer membranes [24,25], electrolytes for fuel cells [26,27], natural product extraction [28] and a wide array of chemical syntheses [29,30,31,32]. The reader is directed to several excellent reviews detailing the use of DES systems in various areas of chemistry and chemical engineering [33,34,35,36,37,38,39].

The use of DES systems in polymer chemistry is gaining increasing attention since the first report of polymerization using a eutectic mixture as far back as 1985 [40]. Two primary themes are emergent: polymerization “in” a DES and the polymerization “of” a DES [41]. DES systems can be used to replace traditional solvents, whereas DES systems where one or more components are polymerizable (often referred to as “polymerizable eutectics” or “deep eutectic monomer solvents”) provide the unique scenario where the monomer plays the dual role of solvent and reactant, whereby mild reaction conditions can be exploited for the polymerization to proceed. This is particularly appealing for monomers that are solids at room temperature, as the preparation of a polymerizable eutectic may facilitate their solvent-free polymerization as a viscous liquid at room temperature. It is critical to note that part of the appeal of using DES systems is they go far beyond simply solvent replacement—their use provides key benefits such as enhanced reaction kinetics and the production of polymeric materials with improved thermal and/or mechanical properties.

The scope of this review article is to examine not only the use of DES systems in polymer and materials science, but also the benefits their use provides in comparison to traditional solvent systems. A brief introduction to the preparation, physical and chemical properties of DES systems is provided, followed by an evaluation of their role in polymer science across numerous settings: polymer synthesis via various mechanisms (radical, reversible deactivation radical polymerization, ring-opening, anionic, polycondensation and electrochemical), polymer and supramolecular gel synthesis, porous monoliths, and molecularly imprinted polymers for sensing and detection. Wherever possible, a comparative focus is applied to equivalent syntheses using current (conventional) approaches, highlighting the appeal and diversity of DES systems.

### 1.1. Preparation and Classification of DES Systems

One of the most appealing aspects regarding the use of DES systems is the simplicity of their preparation from typically inexpensive, abundant starting materials. DESs are regularly synthesized from the heating stirring of two or more solid starting materials [42,43], forming a clear, viscous homogeneous liquid at the eutectic composition. Other methods include grinding [43] (mixing and grinding solid components until a clear liquid forms), evaporation [44] (dissolving all starting components in water followed by subsequent removal of water via evaporation at reduced pressure) and freeze drying [45] (formation of an aqueous solution of all system components followed by freeze drying). Among them, heating and stirring below the melting points of the individual components is arguably the most convenient.

**Figure 1:**
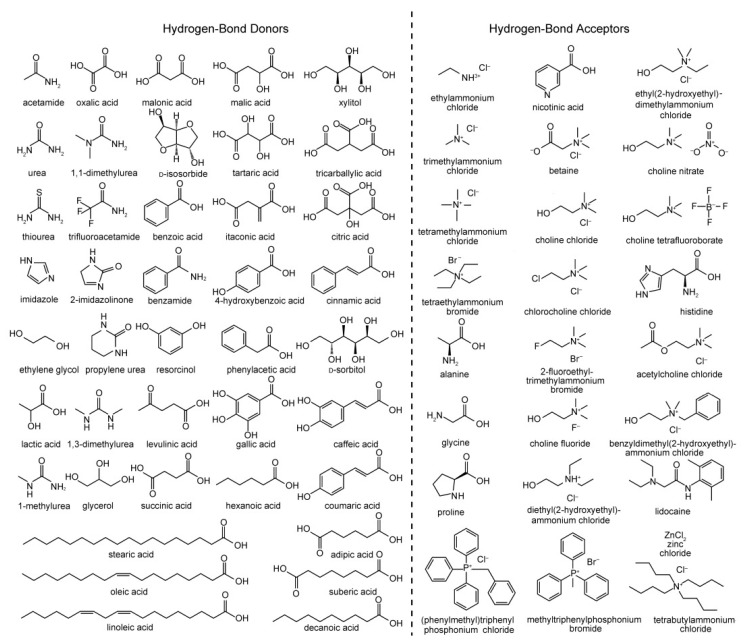
Common hydrogen bond donors (HBDs) and acceptors (HBAs) used in the preparation of various deep eutectic solvent (DES) systems. Reproduced with permission from [10], Copyright 2013, John Wiley and Sons.

A DES system is a mixture that has a significant depression of melting point relative to the melting point of the components, often by well over 100 °C [10]. The first reported DES by Abbott et al. was based on a 2:1 mixture of urea (melting point 133 °C) and choline chloride (ChCl; melting point 302 °C), which possessed a melting point of only 12 °C [9]. This combination of a hydrogen bond donor (HBD) and hydrogen bond acceptor (HBA) mixed at the eutectic composition is particularly versatile given the wide variety of potential compounds that can fulfil these roles; examples of common HBDs and HBAs used for creating DES systems are shown in Figure 1. DES systems are supramolecular in nature, their physical and chemical properties reliant on the association between the HBD and HBA through an extensive hydrogen bonding network. The freezing point depression observed is driven by hydrogen bonding preventing effective crystallization of the components of the mixture, where increased hydrogen bonding strength of the components results in a greater decrease in the melting/freezing point.

DES systems are typically classified into four categories (Types I–IV, respectively) depending on the type of components used [11]. Most DES systems (Types I–III) are based on quaternary ammonium, phosphonium or sulfonium salts with appropriate counterion (a Lewis base such as a halide) mixed with a relevant Lewis acid. Types I and II use non-hydrated (e.g., ZnCl_2_ [46]) and hydrated (e.g., CrCl_3_·6H_2_O [47]) metal halide salts, respectively, while Type III DES systems involve mixing quaternary ammonium salts with common HBDs such as alcohols, amines, and carboxylic acids such as those shown in Figure 1. Type IV eutectics combine metal halide salts with hydrogen bond donors. Type III DES systems are the most widely studied [48], in particular those based on ChCl as HBA as it is cheap, biodegradable, and cost-effective for large-scale use. Furthermore, the wide variety of amines, amides, alcohols and carboxylic derivatives available as HBDs, including those from bio-derived or natural sources, provide great potential for forming DES systems with tailored properties. DES systems can also formed from the mixture of HBDs, which can be termed “non-ionic” deep eutectic solvents [49].

### 1.2. Physical and Chemical Properties of DES Systems

The properties of DES systems can be readily changed depending on the method of preparation and the nature and purity of the participating components, such as: the chemical composition of the quaternary ammonium compound and HBD, the molecular size of the mixed species, their molar ratio, water content (if applicable), and temperature [50]. This makes DES systems particularly flexible in contrast to ionic liquids, which are essentially fixed in their composition. DES systems exhibit unusual solvation properties [9] and because of their tuneable composition, can be exploited in extraction and separation technologies for their potential to dissolve a wide variety of compounds. The nature of the components used to prepare DES systems typically renders them highly polar and hydrophilic; as a result, polar solvents such as water and methanol are miscible with commonly reported DESs, while common non-polar solvents such as toluene, hexane, diethyl ether are immiscible. Hydrophobic DES systems have emerged recently [51,52,53]; however, these are significantly less common. The ionic nature of most DES systems enables metal salts to readily dissolve, which has seen electrodeposition [54,55] and metal plating as some of the most common applications of these solvent systems [11].

Arguably the most striking physical property of DES systems is their very high viscosity (often >>100 cP at 298 K) in comparison to traditional small molecule solvents. The extensive hydrogen bonding network formed during DES synthesis results in the lower mobility of free species within the DES structure, resulting in the high viscosity observed [56,57]. This high viscosity is actually advantageous in many areas of polymer synthesis, discussed in Section 2, despite the challenges presented with respect to manual handling. Despite most DES systems being based on ionic components, most exhibit relatively low ionic conductivities (<2 mS cm^−1^), as a result of the restricted mobility of ionic species due to their high viscosity. Most DES systems exhibit higher densities than water, which can be adjusted based on the composition of the mixture [57]. The thermal stability of DES systems is typically superior to their individual components, as evaluated by thermogravimetric analysis [58,59].

## 2. DES Systems in Polymer and Materials Science

The wide diversity of combinations of HBDs and HBAs that are suitable to generate DES systems has seen their widespread use in various aspects of polymer synthesis. As discussed in Section 1, polymerizable eutectics (i.e., where one or more components is polymerizable, most typically the HBD) are regularly common given the number of monomers bearing amine, amide, hydroxyl or carboxylic acid groups. Polymerizable eutectics are also excellent solvents for effectively dispersing additives (e.g., carbon nanotubes) at high concentration to generate polymer composites in one step. Common DES systems have been used to replace traditional polar solvents in polymer synthesis; however, more hydrophobic monomers (e.g., styrenics and hydrophobic methacrylates) often do not dissolve in pure DES systems and require additional co-solvents. The below sections detail advances in both areas (polymerizable eutectics and DES systems as alternative solvents) grouped by polymerization mechanism or material class.

### 2.1. Mechanisms of Polymerization

#### 2.1.1. Free-Radical Polymerization

**Figure 2:**
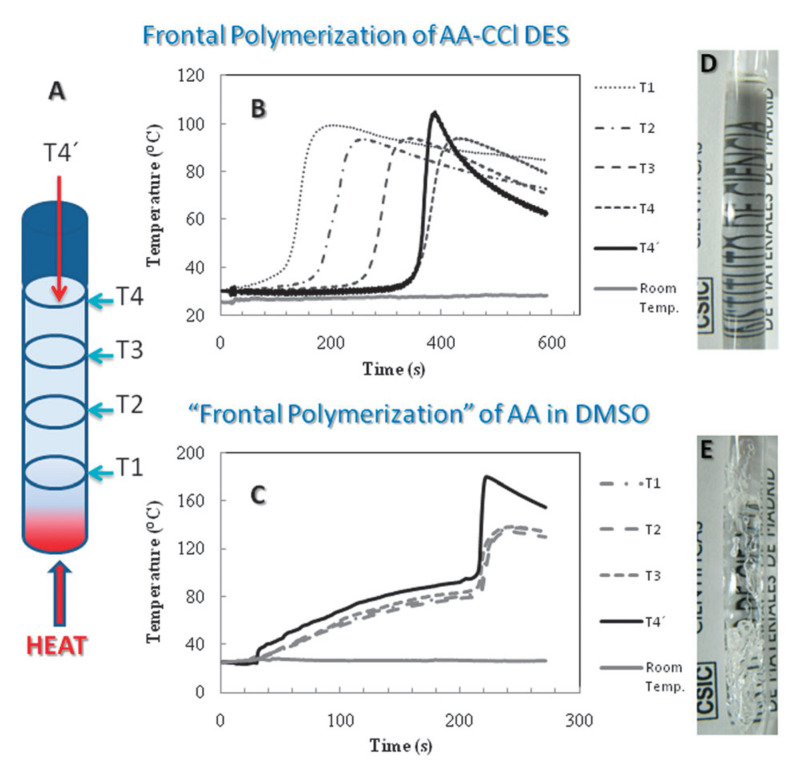
Frontal polymerization using a polymerizable eutectic. (**A**) schematic of the frontal polymerization where T1–T4′ represent various positions along the column where the temperature was monitored (**B**) The temperature profile of the polymerization front in an AA:ChCl DES versus (**C**) the temperature profile of polymerization of acrylic acid (AA) in DMSO, (with digital photographs of the resulting gels (images (**D**) and (**E**) respectively). Reproduced from Ref. [63] with permission from The Royal Society of Chemistry, copyright 2011.

The free-radical polymerization of vinyl monomers in DES systems has been relatively well studied, primarily in the context of frontal polymerization [60,61,62,63]. Mota-Morales et al. [63] first reported polymerizable eutectics based on acrylic (AA) or methacrylic (MAA) acid mixed with ChCl, both of which were liquids at room temperature. The high viscosities of these polymerizable eutectics (115 and 193 cP, respectively, in this work) are ideal for the stabilization of frontal polymerizations, which were performed using benzoyl peroxide as initiator and ethylene glycol dimethacrylate (EDMA) as cross-linker in a column format. A remarkable contrast was seen between the polymerizable eutectic and the equivalent process in DMSO; the polymerization front through the column was smooth and controlled in the case of the polymerizable eutectic, whereas in DMSO the front was uncontrolled with a rapid exotherm (Figure 2). Replacing ChCl with a heat sink (talc), hydrogen bonding diluent (lauric acid) and phase change material (stearic acid) were all unsuccessful in sustaining a successful polymerization front [60], indicating the positive influence ChCl had on polymerization. The equivalent approach was also used to load the anaesthetic lidocaine hydrochloride as the ammonium salt in a polymerizable eutectic with acrylic acid [64]. The controlled release of lidocaine hydrochloride from the polymer monolith over a 48 h timeframe was demonstrated, highlighting potential pharmaceutical applications. Furthermore, nitrogen-doped carbon nanotubes were successfully dispersed within an AA:ChCl eutectic up to a loading of 1% *w*/*w* relative to monomer, enabling the synthesis of macroporous monoliths that were otherwise non-porous in the absence of nanotubes [62]. This was attributed to the strong interaction between AA and the nanotubes, resulting in a homogeneous coating of polymer as confirmed by high-resolution TEM and calorimetry.

Photoinitiators have also been employed to facilitate the free-radical polymerization of monomers in DES systems, in addition to polymerizable eutectics, under particularly mild reaction conditions. Fazende et al. [65] highlighted the impact of polymerizable eutectics with respect to accelerated reaction kinetics via photopolymerization. Using the common photoinitiator diphenyl(2,4,6-trimethylbenzoyl) phosphine oxide (TPO) at a loading of 0.1 mol%, acrylic acid (AA) and methacrylic acid (MAA) showed no polymerization within 90 min via visible light irradiation. In contrast, AA:ChCl and MAA:ChCl eutectics displayed rapid polymerization kinetics as monitored by FTIR spectroscopy. The authors demonstrated that the increased viscosity was alone not the sole reason for the elevated reaction rate, but also the polarity of the DES. This was shown through the photopolymerization of MMA in the presence and absence of a non-reactive DES (isobutyric acid:ChCl), where a five-fold increase in the polymerization rate was observed when a DES was used. A further example of photoinitiated DES systems was from the group of Mecerreyes [66], who prepared 2-cholinium bromide methacrylate (ChBrMA) via the quaternization of (2-dimethylamino)ethyl methacrylate with 2-bromoethanol, followed by the formation of polymerizable eutectics via mixing with citric acid or amidoxime. These polymerizable eutectics were clear viscous liquids at room temperature, enabling photopolymerization with the UV initiator 2-hydroxy-2-methylpropiophenone to generate poly(ionic liquids) for application in CO_2_ capture. 

**Figure 3:**
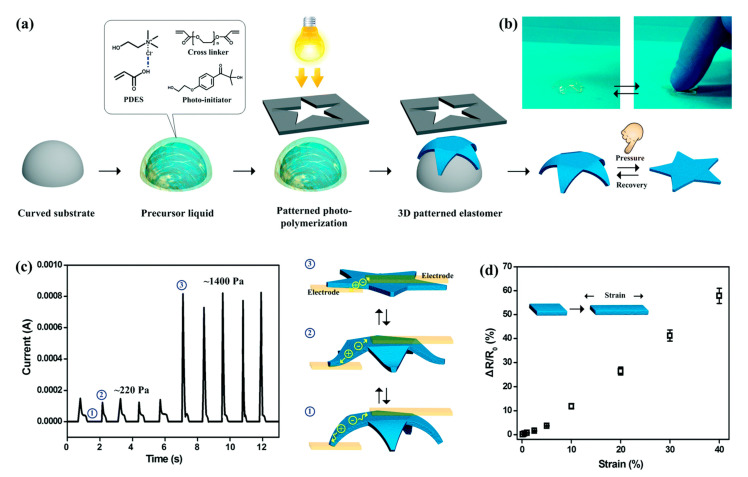
Printed tactile sensors based on AA:ChCl polymerizable eutectics (referred to as PDES in the Figure). (**a**) Overview of photopolymerization and patterning of AA:ChCl eutectic on curved substrates. (**b**) Images of the curved object being reversibly deformed under pressure. (**c**) Measured current through the printed object as a function of applied pressure at various deformation; (**d**) Strain–response plots for stretching the printed object. Reproduced from Ref. [68] with permission from The Royal Society of Chemistry, copyright 2017.

Li Ren’ai et al. [67] have also used photopolymerized AA:ChCl eutectics to prepare conductive paper [67] and strain sensors [68]. Two:one mixtures of AA and ChCl were prepared and photopolymerized in the presence of ethylene glycol diacrylate as cross-linker (1–5% *w*/*w* /relative to DES) and 2-hydroxy-4-(2-hydroxyethoxy)-2-methylpropiophenone (Irgacure 2959^®^) as photoinitiator (2% *w*/*w* relative to DES). The polymerizable eutectics were used as inks and deposited on paper via screen printing into various shapes and patterns, specifically paper origami circuits. Using pre-designed circuit paths and careful integration, on-demand input/output 3D circuits were achieved, showing the potential to construct origami electronics. These polymerizable eutectics were also able to be spot-cured to form optically transparent elastomeric materials of unique shapes that were highly stretchable (up to 150%) and showed good electrical conductivity (0.2 S m^−1^). Tactile and strain-based sensors, such as those for wearable electronics, were demonstrated (Figure 3).

Sánchez-Leija et al. [69] reported the use of the enzyme horseradish peroxidase (HRP) as part of the catalytic system consisting of HRP/hydrogen peroxide/2,4-pentanodione to conduct the free-radical polymerization of acrylamide in aqueous mixtures of ChCl-based DES systems. Polymerizations were conducted in an 80% *v*/*v* DES medium to facilitate suitable viscosities for sample handling, in addition to preservation of the supramolecular structure of the DES. Despite a significantly reduced enzyme activity in this solvent composition relative to phosphate-buffered saline, the room temperature polymerization of acrylamide in this solvent proceeded to ~100% conversion with high molar mass polymer (>100 kDa) formed.

#### 2.1.2. Reversible Deactivation Radical Polymerization (RDRP)

Over the past four decades, RDRP techniques such as nitroxide mediated polymerization (NMP [70]), atom transfer radical polymerization (ATRP [71]) and reversible addition fragmentation chain transfer (RAFT [72]) polymerization have revolutionized polymer science through the ability to create polymers of targeted length and low dispersity, in addition to designer multiblock structures never previously achievable. Despite this, there are only a limited number of reports to date where RDRP has been performed in DES systems. The first demonstrations of RDRP in a DES were from the group of Coelho [73,74], who reported the supplemental activator and reducing agent (SARA) ATRP of various acrylates and methacrylates. Their first report detailed the SARA ATRP of MA using reline (a 2:1 urea:ChCl DES) as a co-solvent—the solvent was 90% *v*/*v* ethanol (10% *v*/*v* reline) due to the immiscibility of MA with reline. Low dispersity polymers (*Đ* < 1.2) of the order of 9 kDa were prepared and chain extension with BA was successfully demonstrated. The DES content within the solvent mixture, while low, enabled excellent recovery of the Cu catalyst (up to 62%) due to in situ phase separation between a polymer-rich phase and a solvent-rich phase. The recovered catalyst was used successfully in two additional SARA ATRP experiments with good performance.

ATRP performed in neat DES systems was subsequently reported by the groups of Coelho [74], Presa Soto [75] and Xue [76]. Coelho’s group used reline as the solvent for the SARA ATRP of 2-hydroxyethyl acrylate (HEA), 2-hydroxyethyl methacrylate (HEMA) and (3-acrylamidopropyl)trimethylammonium chloride (AMPTMA) at 30 °C using sodium dithionite as the SARA agent. Compared to previous experiments with MA, HEA, HEMA and AMPTMA were all miscible with reline which was attributed to hydrogen bonding with the solvent. For the polymerization of HEA, polymers from 9 kDa to 45 kDa were successfully prepared, however the polymer dispersity was >1.5 when targeting a degree of polymerization (DP) > 600 ([Cu(II)] < 200 ppm), indicative of a loss of molar mass control. Chain extension to form block copolymers was best realized with a slow feed of the SARA agent into the reaction mixture; when the SARA agent was added in batch at the start of the reaction a significant fraction of the first block did not undergo chain extension. Quirós-Montes et al. [75] performed the ARGET ATRP of MMA in a 2:1 glycerol:ChCl DES, using tin (II) 2-ethylhexanoate as reducing agent in an air atmosphere. Focusing on green chemistry development, they used a Cu-based metal organic framework as the metal catalyst for successful polymerization. This enabled easy recovery of the Cu catalyst and re-use for subsequent polymerizations (with good performance over six cycles), and less than 0.5 ppb residual copper detected in the final polymeric product.

Wang et al. [76] performed a comprehensive analysis of the ATRP of MMA with FeBr_2_ across a range of DES systems as potential solvents (13 different solvents consisting of Type I, II and III eutectics spanning with melting points from <−70 °C to 48 °C). The polymerizations were conducted in the absence of any additional ligands. Of the Type I DES systems studied, only acetamide:ε-caprolactam was a suitable polymerization solvent—other Type I solvents gave poor solubility of MMA or catalyst precipitation. Across a range of targeted DPs (from 50 to 200 units) at a monomer:solvent ratio of 3:1, polyMMA formed in this DES gave only moderate control of the molar mass distribution with reported dispersities in the range 1.36 < *Đ* < 1.77. Type II (based on thiocyanate salts with an amide (acetamide or urea)) and Type III (quarternary ammonium salts with either glycerol, ethylene glycol or malonic acid) DES systems were able to afford excellent molar mass control of resulting polymers (*Đ* < 1.17); however, these were essentially bulk polymerizations (monomer:solvent = 200:1) where the DES components were acting as ligands for the ATRP process. Cyclic voltammetry was used to establish changes in the reduction potential of Fe^2+^ in the presence of DES systems, highlighting the ability for DES components to serve as ATRP ligands and facilitate the polymerization process.

**Figure 4:**
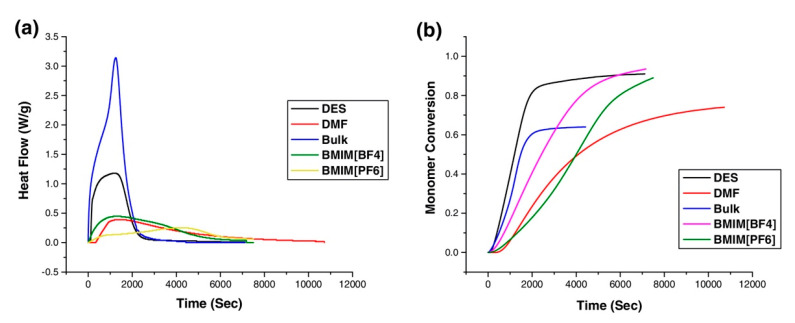
Calorimetric analysis of the reversible addition fragmentation chain transfer (RAFT) polymerization of 2-hydroxyethyl methacrylate (HEMA) in a variety of solvents; 2:1 urea:ChCl (black), DMF (red), bulk (blue) and ionic liquids (green, yellow, purple, respectively). Shown are heat flow vs. time (**a**) and conversion vs. time (**b**) for polymerization at 70 °C. Reproduced with permission from [77], Copyright 2019, John Wiley and Sons.

To date, there are only two reported examples of RAFT polymerization in DES systems [77]. Santha Kumar et al. reported the RAFT polymerization of HEMA in reline (a 2:1 urea:ChCl mixture) at 70 °C mediated by the RAFT agent 4-cyano-4-[(dodecylsulfanylthiocarbonyl)sulfanyl]pentanoic acid (CDTSPA). A range of [monomer]:[RAFT] ratios were studied (from 115 to 345), with high conversion (~90%) after 3 h of polymerization and moderate molar mass control (*Đ* ~ 1.40). Differential scanning calorimetry (DSC) was used to monitor polymerization kinetics and a comparative study between reline and other solvents was performed (dimethylformamide (DMF), bulk polymerization and various ionic liquids). Highlighting the power of DES systems, the polymerization kinetics were significantly greater than equivalent polymerizations in DMF and ionic liquids; higher conversion was achieved in reline than in bulk (Figure 4). Chain extension with MMA enabled the first demonstration of polymerization-induced self-assembly (PISA) in a DES system, giving spherical polymer nanoparticles of diameter 65–70 nm as determined by scanning electron microscopy. Pereira [52] et al. used the bio-sourced 2:1 d,l-menthol:1-tetradecanol hydrophobic DES to successfully perform the RAFT polymerization of styrene, MMA, MA, vinyl acetate and vinyl chloride at 60 °C (42 °C for vinyl chloride), achieving high fractional conversion and excellent molar mass control (*Đ* < 1.13 with the exception of vinyl chloride, where *Đ* = 1.33). The authors also used this solvent for SARA and ARGET ATRP of MA, MMA and styrene with both excellent molar mass control and the ability to recover the polymerization catalyst due to in situ phase separation. The hydrophobic nature of this DES and ability to dissolve hydrophobic monomers greatly expands the potential of DES systems for the successful RDRP of a wide variety of vinyl compounds in the future.

#### 2.1.3. Anionic Polymerization

**Figure 5:**
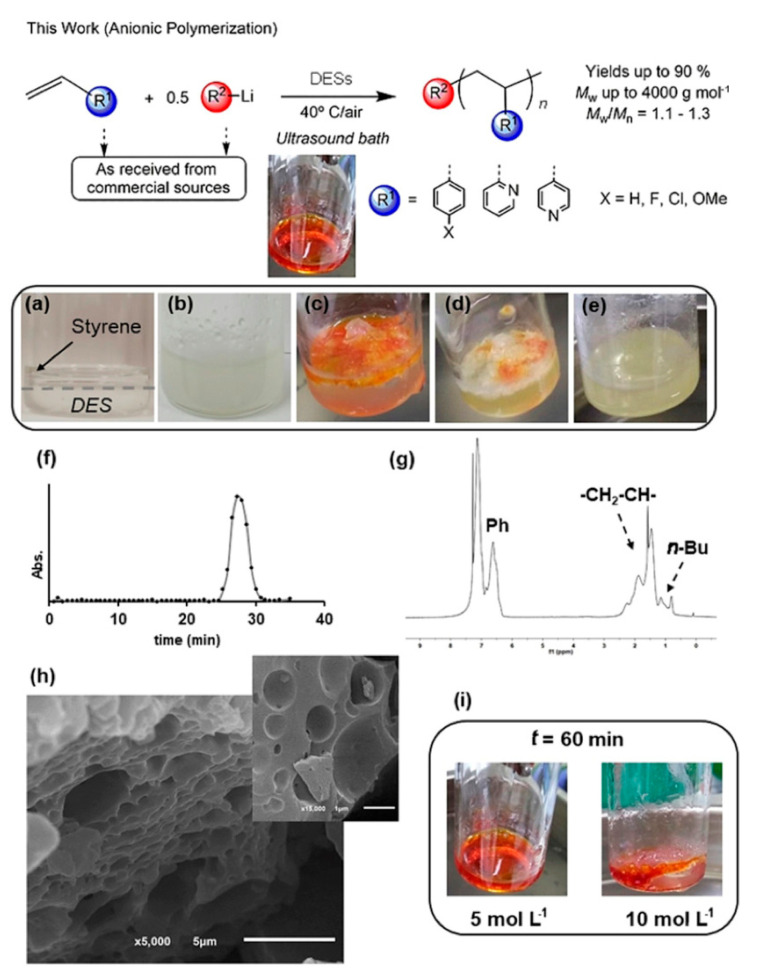
Anionic polymerization of various *p*-substituted styrenes, 2-vinylpyridine and 4-vinylpyridine in a 2:1 glycerol:ChCl DES open to the air. Figures (**a**–**e**): photographs of the reaction ([styrene] = 1 M) before and after ultrasonication, 5, 10 and 20 min after addition of *n*-BuLi. Figures (**f**,**g**): Size exclusion chromatography (SEC) trace and ^1^H NMR spectra of polystyrene formed by this approach. Figure (**h**): SEM image of isolated polystyrene. Figure (**i**): photograph of the reaction after 60 min polymerization at different styrene concentrations. Adapted with permission from [80], Copyright 2019 John Wiley and Sons.

Since the first report of the polymerization of styrene with sodium napthaleneide as initiator by Szwarc in 1956 [78,79], anionic polymerization has been considered one of the primary methods for the preparation of well-defined polymers with low dispersity. Anionic polymerizations are typically initiated by organolithium compounds, which can be sensitive to air, moisture and solvent contaminants. As a result, anionic polymerizations are typically conducted under high vacuum, inert-atmosphere conditions with high purity starting materials, which makes them less easy to implement compared to the RDRP techniques discussed in the previous section. The work of Sánchez-Condado [80] is thus more remarkable, as they demonstrated that anionic polymerization of styrene, various *p*-substituted styrenes, and both random and block copolymers of styrene with 2-vinylpyridine using various organolithium compounds can be conducted in a 2:1 glycerol:ChCl DES at 40 °C in the presence of air to generate polymers of low dispersity (Figure 5). A critical aspect of the work was the ultrasonication of the monomer with the DES to generate a dispersion (given that these aromatic monomers were not soluble in the DES), prior to the addition of the organolithium compound. In this DES, the resulting polystyryl anions were stable towards hydrolysis for up to 90 min, enabling the formation of polymers with *M_n_* ~2–4 kDa with dispersities between 1.1 and 1.3. Further, the anionic polymerization of *p*-chlorostyrene was reported for the first time in this work. While the polymer molar masses in this work were relatively low, the concept of developing simple, green chemistry approaches to facilitate anionic polymerization is particularly appealing.

#### 2.1.4. Ring Opening Polymerization (ROP)

Important commodity polymers such as poly(lactide) (PLA) and poly(caprolactone) (PCL) are commonly prepared via ring opening polymerization (ROP), routinely at high temperatures and under relatively harsh conditions [81]. As a result, greener approaches such as the use of polymerizable eutectics have been explored. This concept was first reported by Coulembier [82] et al., who prepared eutectics based on a 1:1 mass ratio of l-lactide (L-LA) and trimethylene carbonate (TMC); the eutectic had a melting point of 23 °C. The homopolymerization of L-LA was achieved at room temperature using benzyl alcohol as initiator and 1,8-diazabicyclo [5.4.0]undec-7-ene (DBU) as catalyst, respectively. TMC can also undergo ROP, and copolymers of L-LA and TMC were achieved by raising the temperature beyond the *T*_g_ of PLA. L-LA has also been shown to form room-temperature liquid eutectics with ε-caprolactone (CL [83]), which were used to create polymerized high internal phase emulsions (polyHIPEs), discussed in Section 2.3.2.

For the ring opening polymerization of CL, García-Argüelles et al. [84] prepared eutectic or near-to-eutectic mixtures of methanesulfonic acid and guanidine 1,5,7-triazabicyclo[4.4.0]dec-5-ene (TBD) as co-catalysts. This enabled the polymerization of CL at 37 °C in the absence of initiator and solvent generating polymers between 6 and 10 kDa with exceptionally high crystallinity (between 84% and 98%); this level of crystallinity is significantly higher than that possible via high-temperature polymerization in traditional organic solvents (of the order of 65%). Films prepared from poly(caprolactone) synthesized by this approach were shown to support the adhesion and growth of fibroblasts; the absence of volatile solvents is particularly appealing in the context of polymers for biomaterials applications.

#### 2.1.5. Polycondensation

**Figure 6:**
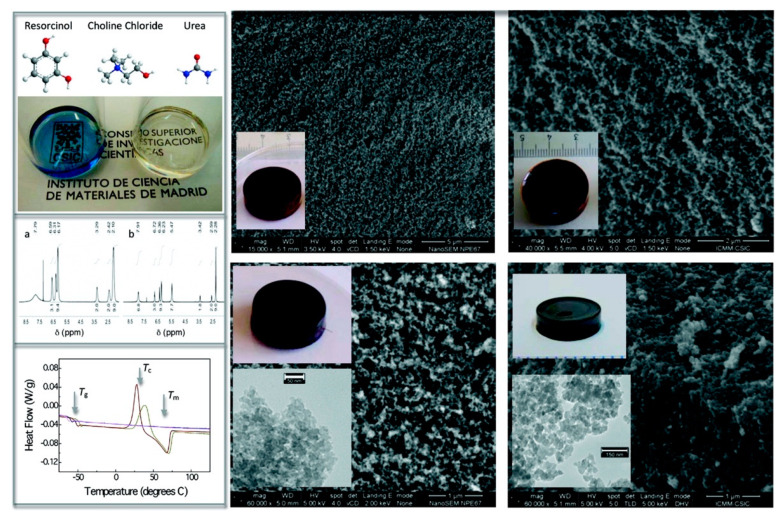
Preparation of porous carbon monoliths via resorcinol-formaldehyde polycondensation from polymerizable eutectics. Left panel: preparation and accompanying characterization of resorcinol:ChCl and resorcinol:ChCl:urea polymerizable eutectics; the blue sample contains urea. Right panel: SEM images and digital photographs of polymer resins (**top**) and monolithic carbons (**bottom**) prepared from these eutectics. Also provided are TEM insets of the carbon monoliths. Adapted with permission from [86], Copyright 2010 American Chemical Society.

The group of del Monte has made significant advances in the design of materials via polycondensation in DES systems [85,86,87,88,89,90]. The polycondensation of resorcinol (benzene-1,3-diol) with formaldehyde was performed in in an ethylene glycol:ChCl DES using water as a comparative solvent [85], followed by washing and carbonization to form monolithic carbon-based materials. The powerful nature of the DES solvent enabled high loadings of resorcinol and formaldehyde in the initial sol, resulting in a greater degree of condensation in the resulting polymer. In comparison to gels prepared in water, the resulting carbon monolith had significantly higher surface area (120–170 m^2^ g^−1^ compared to ~7 m^2^ g^−1^). The use of DES systems enabled dispersion of multiwalled carbon nanotubes at high loading (20% *w*/*w* relative to solvent) into the initial sol that was not possible via aqueous solution, further increasing the surface area of the resulting carbon monolith (~350 m^2^ g^−1^). Exploiting the polymerizable eutectic concept, DES systems incorporating resorcinol as HBD were developed [86], whereby resorcinol was mixed with ChCl (or urea and ChCl) to generate clear viscous liquids with melting points in the range 68–87 °C (Figure 6). Polycondensation via the addition of formaldehyde and subsequent carbonization was performed to generate monolithic carbons with even greater surface area (>500 m^2^ g^−1^) than those described above. Interestingly, these monoliths displayed mesoporosity with narrow pore size distribution, that was able to be varied through the presence of absence of urea within the starting sol (23 nm diameter pores with urea present, compared to ~10 nm diameter pores in the absence of urea).

Other polymerizable eutectics have been explored for polycondensation and subsequent carbonization to create a variety of carbon monoliths. These include ternary eutectics based on resorcinol:3-hydroxypyridine:ChCl [88,89], resorcinol:3-hydroxypyridine:tetraethylammonium bromide [90] and resorcinol:4-hexylresorcinol:tetraethylammonium bromide [91]. These eutectics were used for the base catalyzed polycondensation with formaldehyde and were designed for specific purposes; for example, the use of 3-hydroxypyridine enabled in situ incorporation of nitrogen into the monolith structure at loadings as high as 5% mol/mol relative to carbon [88]. These N-doped monoliths were shown to be excellent materials for CO_2_ adsorption (up to 3.3 mmol CO_2_ g^−1^) with selective adsorption of CO_2_ over N_2_ that compared favorably to many zeolites and metal-organic frameworks [90]. Polycondensation of formaldehyde using this eutectic in the presence of iron acetate resulted in the creation of high surface area monoliths with graphitic character and very high electrical conductivity (up to 31 S cm^−1^ [89]). DES systems have also been prepared as catalysts for the polycondensation of furfuryl alcohol to ultimately produce porous carbon electrodes [87], as well for the effective solubilization of lignin to effectively incorporate biomass fillers into phenol-formaldehyde resins [92].

Other classes of polymers have been prepared via polycondensation in DES systems. Poly(diol-*co*-citrate) polyesters were prepared by Serrano et al. [93] and García-Argüelles et al. [94] through the development of DES systems based on 1,8-octanediol. Serrano et al. [93] used 1,8-octanediol and lidocaine in various ratios (1:1 through to 1:3) to yield viscous liquids with melting points as low as 41.8 °C. Polymerization at 90 °C was performed through the addition of citric acid, yielding a cross-linked elastomeric material. Lidocaine was specifically chosen both as HBA and as an active pharmaceutical ingredient that could be incorporated at high loadings for subsequent controlled release. The same class of polymers where prepared from DES systems combining 1,8-octanediol with various chloride or bromide salts to yield polymeric materials with antibacterial properties [94].

#### 2.1.6. Electrochemical Polymerization

DES systems find application in the electrochemical polymerization of important classes of monomers for the preparation of electrically conductive polymers. Fernandes et al. [95,96] demonstrated the applicability of various DES systems for the electrochemical polymerization of aniline. Reline, ethaline and glyceline (2:1 mixtures of urea, ethylene glycol and glycerol with ChCl, respectively) were used as solvents for the electrochemical deposition of poly(aniline) onto glassy carbon electrodes. Polymerization in reline was relatively slow in comparison to ethaline and glyceline; furthermore, the modified electrodes prepared from reline were the least electroactive which was attributed to a sparse distribution of nanoparticles of poly(aniline) across the electrode surface when visualized by SEM. The relative order of electroactivity of the polyaniline films showed an inverse correlation with the freezing temperature and viscosity of the DES. Reline, with the highest freezing point and viscosity (and subsequently lowest conductivity) gave the poorest films, with ethaline the best. The conductivity of the films prepared from ethaline and glyceline were as high as 63.8 S cm^−1^, over 10 times greater than poly(aniline) films prepared from aqueous solution. The same group also used propeline (a 2:1 mixture of 1,2-propanediol and ChCl) as solvent for poly(aniline) synthesis.

Brett’s group has reported the synthesis of poly(3,4-ethylenedioxythiophene) (PEDOT) by electrochemical means in DES systems [97,98]. Electropolymerization of 10 mM solutions of EDOT was performed in ethaline, glyceline and reline in the presence of 4 M HClO_4_ to form uniform films on glassy carbon electrodes. The presence of HClO_4_ was critical as EDOT polymerization in pure DES systems was shown to be unsuccessful. Comparative experiments were performed using aqueous 0.1 M poly(sodium styrene sulfonate) and aqueous 4 M HClO_4_ solutions as solvents for electrodeposition. In contrast to poly(aniline) synthesis discussed above, reline was considered the best DES for PEDOT synthesis, with well-defined anodic and cathodic peaks during synthesis and a compact surface morphology of the polymerized films. Electrochemical quartz crystal microbalance (EQCM) studied revealed an 11-fold decrease in deposited mass of PEDOT on the electrodes when using reline in comparison to aqueous systems, giving rise to thin, non-rigid and porous films that have advantageous sensing characteristics. The PEDOT-coated electrodes from reline had the highest capacitance of all films prepared and were also the most sensitive with respect to amperometric detection of ascorbate oxidation (245 μA cm^−2^ mM^−1^) at an operating voltage of 0.0 V. The simultaneous detection and quantification of ascorbic acid, uric acid and dopamine using PEDOT-coated electrodes from reline was also demonstrated.

Hosu et al. [99] used the DES system ethaline as the solvent for the electrodeposition of poly(methylene blue) onto glassy carbon electrodes. Methylene blue (MB) was first dissolved in 1 M NaOH due to its limited solubility in ethaline, followed by the addition of ethaline and subsequent neutralization to form to form a 9:1 ethaline:aqueous mixture. The scan rate was the critical parameter with regards to forming nanostructured polymer films that were suitable for electrochemical sensing. Films prepared from DES had higher redox currents and lower charge transfer resistance than films prepared directly from aqueous solution, highlighting the benefit of using these solvents. The electrodes were used to monitor the oxidation of ascorbate, to which the films prepared from a DES had more than 10-fold increased sensitivity (350 μA cm^−2^ mM^−1^ vs. 23 μA cm^−2^ mM^−1^) with comparable limit of detection compared to equivalent aqueous preparation.

While not related to electrochemical polymerization, it is worth noting that DES systems have found utility in the preparation for the creation of lithium metal batteries [100]. Jaumaux et al. prepared eutectics based on lithium bis(trifluoromethanesulfonyl)imide (LiTSFI) and *N*-methylacetamide, with fluoroethylene carbonate as an additive. This solvent was used for the radical polymerization of pentraerythritol tetraacrylate (PETTA) and 2-(3-(6-methyl-4-oxo-1,4-dihydropyrimidin-2-yl)ureideo)ethyl methacrylate to generate a crosslinked polymer matrix that effectively “trapped” the DES electrolyte in a pseudo-solid state form to reduce electrolyte leakage from the prepared electrodes, which is a significant safety issue in modern lithium batteries.

### 2.2. Preparation of Hydrogels

#### 2.2.1. Polymer Hydrogels

DES systems have been used in various contexts for the preparation of a wide variety of polymeric hydrogels. These include the use of polymerizable eutectics (where one or more components of the eutectic is a polymerizable compound), either in bulk or as a frontal polymerization, or as an alternative solvent system. In comparison to traditional approaches to hydrogel synthesis, gels prepared from DES systems typically exhibit different physical or chemical properties which are discussed below.

Several examples of gels prepared from polymerizable eutectics have been reported. Bednarz et al. [101,102] used a 1:1 itaconic acid (IA):ChCl DES to prepare cross-linked poly(itaconic acid–*co*–*N,N’*-methylenebisacrylamide (BIS)) hydrogels. A comparative study for gel synthesis in water was performed using potassium persulfate (KPS) as initiator at 65 °C, which proceeded to completion within 2 h in the eutectic, but only ~50% conversion in water. The higher polymerization rate in the polymerizable eutectic was accompanied by significantly greater gel fraction (91% vs. 33%) and reduced degree of swelling when immersed in water (11 vs. 66 g g^−1^), indicative of a significant increase in cross-linking density within the gel. In similar work [103], the same authors demonstrated the synthesis of macroporous xerogels in the absence of any surfactant through the addition of low molecular weight poly(ethylene glycol) (PEG; either 1500 or 3000 Da) at a loading of 9% *w*/*w* into a 1:1 IA:ChCl eutectic mixture, followed by polymerization and cross-linking. The inclusion of PEG as resulted in phase separation and the generation of a regular void structure within the gels, with void dimensions of the order of 1–4 µm and significant reduction in specific surface area compared to gels prepared in the absence of PEG. The gels prepared in this work were shown to remove Cu^2+^ from water (up to 83 mg g^−1^) as a potential application of these materials, with suggested other applications including solid supports for immobilization of microbial cells and/or enzymes, or as stationary phases in chromatography of biomolecules.

**Figure 7:**
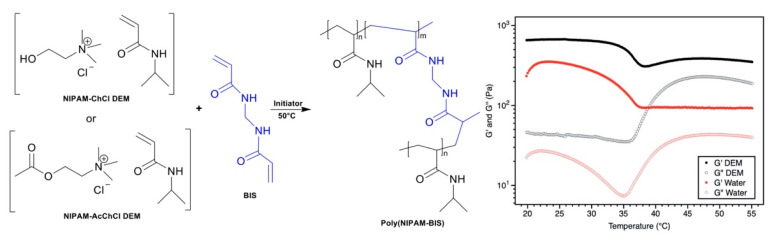
Thermoresponsive hydrogels via 3:1 *N*-isopropylacrylamide (NIPAM):ChCl/AcChCl polymerizable eutectics. The resulting hydrogels exhibited a significant increase in shear modulus compared to equivalent gels prepared in water. Adapted from Ref [104] with permission from The Royal Society of Chemistry, copyright 2020.

Our group [104] recently reported the preparation of cross-linked thermoresponsive poly(*N*-isopropylacrylamide-*co*-*N,N’*-methylenebisacrylamide) hydrogels prepared directly from polymerizable eutectics based on *N*-isopropylacrylamide (NIPAM) and various choline salts (Figure 7). NIPAM-based eutectics with a 3:1 composition were shown to possess low melting points of 39 °C and 15 °C, respectively, when prepared with ChCl and acetylcholine chloride (AcChCl), respectively, which were subsequently polymerized and cross-linked via the addition of potassium persulfate and BIS, respectively. In comparison to hydrogel synthesis in water, the use of a polymerizable eutectic provided several advantages: a six-fold increase in polymerization kinetics was observed, with a resultant gel fraction close to 100%. Furthermore, rheological analysis of these gels indicated an increase in the shear storage and loss moduli at 293 K by a factor of 2.8 and 2.1, respectively. This was attributed to a much higher cross-linking density in the gels prepared from polymerizable eutectics, which was further confirmed by the reduced swelling capacity of these gels relative to those prepared in water. The thermoresponsive nature of these gels above and below the lower critical solution temperature (LCST) of polyNIPAM was also broad and continuous, as opposed to the more discontinuous volume change for gels prepared in water.

Frontal polymerization (see also Section 2.1.1) has been used as another method to prepare hydrogels from polymerizable eutectics, in contrast to the bulk polymerization methods described above. Jiang et al. [105] fabricated macroporous polyacrylamide hydrogels via the frontal polymerization of a polymerizable eutectic consisting of acrylamide (AM) and ChCl; the lowest recorded melting point was a 2:1 AM:ChCl eutectic at 32 °C; this composition had a particularly high viscosity of 130 cP at 35 °C. Using KPS as an initiator and BIS as cross-linker, frontal polymerization was performed in a 100 mm glass tube (internal diameter 13 mm) to form hydrogels. In contrast to the equivalent solution polymerization in DMSO, the use of the polymerizable eutectic gave a significant increase in the velocity and temperature of the polymerization front. The resulting gels were further studied with regards to their swelling response in water, with gels prepared from polymerizable eutectics showing faster swelling/de-swelling kinetics. Interestingly, the equilibrium swelling capacity of gels prepared from a ChCl eutectic was almost twice as large as the same gel prepared from DMSO, displaying the reverse trend of our work and that of Bednarz discussed above.

In addition to polymerizable eutectics, the group of Prasad has investigated the preparation of polymeric gels using DES systems as replacements for traditional solvents. Using a ChCl:orcinol 1:1.5 DES, the self-polymerization of HEMA was performed [106] to form highly stretchy gels (stretching to over thirty times their initial length). Only HEMA was shown to self-polymerize in this context, with NIPAM and vinyl acetate showing no polymerization. The DES was shown to be critical to initiate polymerization without further exogeneous initiator, as formulations with the traditional radical inhibitor hydroquinone showed no polymerization. The resulting gels were shown to be suitable polymeric supports for supercapacitors. Showing the versatility of this approach, the self-polymerization of HEMA in a ChCl:fructose 2:1 DES [107] enabled the synthesis of hydrogels with the in situ loading of the anti-inflammatory drug indomethacin into the gel structure. The gels showed good release of indomethacin (~90%) at physiological pH whilst showing no cytotoxicity towards human A549 cell lines. Crosslinked poly(HEMA-*co*-PEGDA) gels have also been prepared from 2:1 EG:ChCl DES systems for incorporation into supercapacitors [108], acting as a gel electrolyte prepared in the absence of hazardous organic solvents.

#### 2.2.2. Self-Assembled/Supramolecular Gels

In addition to polymer-based gels described in the previous section, a further important class of gels are self-assembled or “supramolecular” gels that form via the self-assembly of low molar mass compounds [109]. Supramolecular gels are often formed through an extensive hydrogen-bonding network formed between the gel components [110]; given the critical role hydrogen bonding plays in DES formation, there is potential significant overlap between these two research areas and represents an emerging area of potential materials research. Examples of supramolecular gels prepared from DES systems are discussed below.

One of the first, and perhaps simplest supramolecular gels from a DES was reported by Florindo et al. [53]. A 1:4 ratio of sodium dodecanoate and decanoic acid was shown to form a clear, viscous liquid with a melting point of 22 °C. Despite the components being relatively hydrophobic, the addition of water did not result in phase separation but instead the inclusion of water within the structure, yielding weak shear-thinning gels. These gels also exhibited a thermoresponsive character as a function of the water content.

Smith’s group recently demonstrated the formation of supramolecular “eutectogels” through the self-assembly of 1,3:2,4-dinbenzylidine-d-sorbitol (DBS) in a wide variety of DES systems based on ChCl with various alcohols and urea [111]. DBS was added to various DES systems at a loading of 5% *v*/*v*; gelation occurred either via a heating-cooling cycle or via ultrasonication, giving rise to nanofibrillar networks of DMBS as confirmed via X-ray diffraction and scanning electron microscopy. The gels were shown to have ionic conductivities comparable to the conductivity of the DES from which the gel was prepared; gels prepared from ethylene glycol, propylene glycol and 1,3-propanediol had room temperature ionic conductivities >1 mS cm^−1^, making them potentially useful as gel electrolytes for various applications. The gels were also tolerant to Li^+^, Mg^2+^ and Ca^2+^ additives.

**Figure 8:**
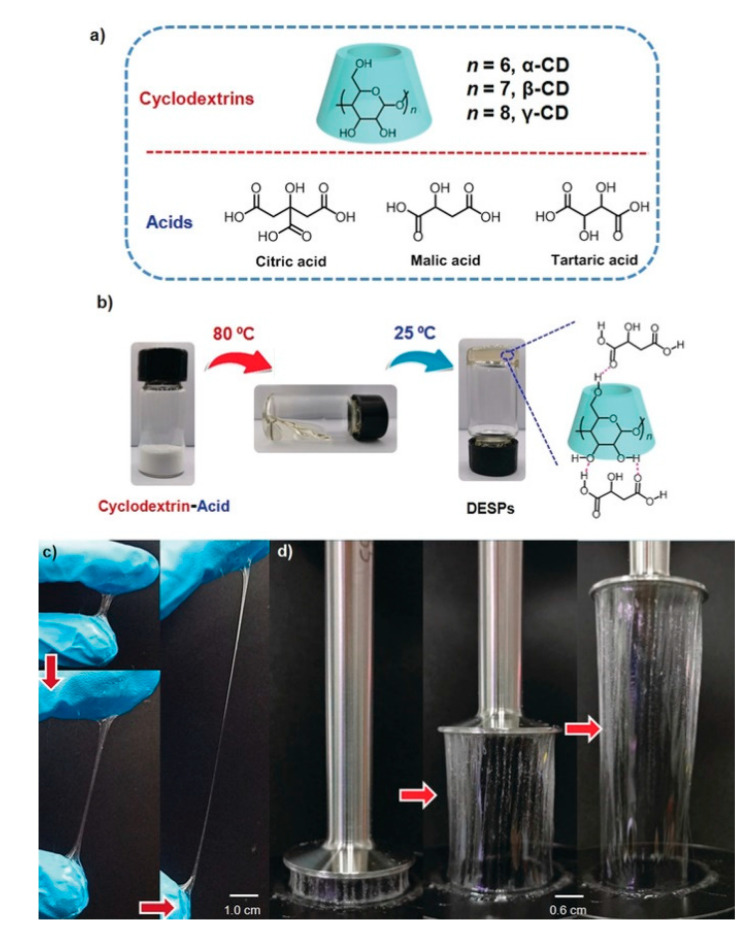
Images (**a**,**b**): supramolecular gels (“Deep eutectic supramolecular polymers”) based on the physical mixing of various cyclodextrins with natural acids. Images (**c**,**d**): drawn fibers and films from these gels cast from gloves and rheology plates. Reproduced with permission from [112], John Wiley and Sons.

Wu et al. [112] prepared what they termed “deep eutectic supramolecular polymers” based on mixing and heating cyclodextrins with various acids (citric, malic and tartaric acid) to form high-viscosity gels with unique mechanical properties. The resulting gels had glass transition temperatures <−20 °C and were formed via hydrogen bonding between the -OH groups present on the cyclodextrin and the various acids; methylated cyclodextrins demonstrated no capability to form gels. These gels were able to be cast and extruded into fibers and films (Figure 8) and also remarkably showed adhesion to a variety of substrates (paper, glass, textile and silicon) with impressive load-bearing capability. The biological relevance of the components of these gels enabled their demonstration as biological glues, enabling adhesion of two pieces of porcine skin. The adhesive strength of these gels was shown to be comparable to well-known catechol-based adhesives.

### 2.3. Porous Polymer Monoliths

Porous polymer monoliths are typically prepared by one of two main methods, namely (i) the polymerization of monomer(s) and crosslinkers in the presence of a porogen, typically a non-solvent to elicit nano- and/or micro-phase separation, and (ii) the polymerization of high internal phase emulsions (HIPEs), often referred to as polyHIPES. In both cases, a porous material typically with interconnected voids are produced that find application in various settings [113]. Given the rise of DES systems; eutectics of various composition have found use in the preparation of monoliths via various approaches. The ability to tune specific solvent properties, both chemical and physical, make DES systems particularly appealing in preparing monoliths with tuneable surface area and surface functionality.

#### 2.3.1. Synthesis via Porogens

Ndizeye et al. reported [49] the use of various *N*-methylated non-ionic deep eutectic solvents as porogens in the synthesis of a variety of crosslinked polymer monoliths. Specifically, acetamide:*N*-methylacetamide (AA-NMA), *N*-methylacetamide:*N*-methylurea (NMA-NMU) and *N*-methylacetamide:*N,N′*-dimethylurea (NMA-NN′DMU) DES systems were prepared, all possessing melting points below 15 °C. These solvents were used in the polymerization of either HEMA or methacrylic acid (MAA), using divinylbenzene (DVB) or 1,4-bis(acryloyl)piperazine (BAP) as cross-linkers in a 12:55 mole ratio of monomer to crosslinker. In all cases, the monoliths prepared from DES systems had a higher specific surface area (up to ~533 m^2^ g^−1^) than the same monoliths prepared in traditional solvents such as toluene, acetonitrile and water.

DES systems, both polymerizable and non-polymerizable, have found use as porogens for the synthesis of monolithic columns for chromatographic applications. Wang et al. [114] used a IA:ChCl 5:1 polymerizable eutectic to generate polymer monoliths within a surface-modified poly(ether ether ketone) tube with 0.508 mm internal diameter via copolymerization with ethylene glycol dimethacrylate (EDMA), using isopropanol as the non-solvent. These monoliths were used for solid-phase microextraction (SPME) and separation of the non-steroidal anti-inflammatory drugs ketoprofen, flurbiprofen and diclofenac sodium from aqueous solution. The group of Liu has also reported the use of ChCl:propylene glycol DES systems (in conjunction with room temperature ILs) as porogens for the development of poly(butyl methacrylate-*co*-ethylene glycol dimethacrylate) monoliths with incorporated carbon nanotubes [115] or MCM-41 molecular sieves [116]. Carbon nanotubes were able to be successfully dispersed into the pre-polymerization mixture without oxidative cutting and with no observed precipitation. Both monoliths exhibited high column efficiency (over 200,000 plates m^−1^ in both cases) and good separation of mixtures of alkylphenones, alkylbenzenes, non-steroidal anti-inflammatory drugs (NSAIDs) and polycyclic aromatic hydrocarbons.

#### 2.3.2. Polymerized High Internal Phase Emulsions (PolyHIPEs)

Another method for the preparation of porous polymer monoliths is the polymerization of the continuous phase of an HIPE. HIPEs are concentrated emulsions where the dispersed phase is >74% *v*/*v*, stabilized most commonly by small molecule or polymeric surfactants. The close packing of the emulsion droplets and subsequent polymerization of the continuous phase (and removal of the droplet phase) results in the formation of a cellular monolithic structure, typically with interconnected pores [117,118]. PolyHIPEs have found numerous applications in areas such as biomaterials [119], separation science [120] and catalysis [121].

The group of Mota-Morales has used DES systems as the dispersed phase for the preparation of polyHIPES [122,123,124]. In their initial work [122], a 2:1 urea:ChCl DES was prepared (80% *v*/*v*) and mixed with an organic continuous phase (20% *v*/*v*) consisting of stearyl methacrylate and EDMA as cross-linker. This was followed by vortex mixing to generate an HIPE stabilized by the non-ionic polyol surfactant Cithrol^®^ at a loading of 7–10% *w*/*w* relative to the total mass of the emulsion. The resulting stable emulsions were polymerized at 50 °C for 36 h through the addition of AIBN as radical initiator. The equivalent all-acrylic HIPEs were also prepared through the use of lauryl acrylate and 1,4-butanediol diacrylate, respectively. Following polymerization, the washing of the resulting monoliths resulted in the excellent recovery of the DES components (up to 95%), highlighting the sustainability of this synthetic approach. SEM and BET analysis of the resulting monoliths demonstrated interconnected macroporosity with voids in the size range of 6–55 µm. The same approach was used to prepare composite monoliths by loading either nitrogen-doped or carbonyl-functionalized carbon nanotubes into the DES dispersed phase of a methacrylic or styrenic HIPE [123]. In this case, the carbon nanotubes contributed to HIPE stabilization while also rendering the final monolith significantly more hydrophobic (based on water contact angle measurements) at nanotube loadings as low as 0.04% *w*/*w* with respect to the continuous phase. These materials were demonstrated as potential adsorbents for fuels such as diesel, biodiesel, gasoline and hexane, with high sorption capacities.

The same group has investigated different DES systems for the preparation of styrenic polyHIPEs [124]. Styrene and divinylbenzene in a 10:1 mole ratio were used as the continuous phase (20% *v*/*v*) for the preparation of HIPEs where the dispersed phase was a ChCl-based DES (either urea, glycerol or ethylene glycol, mixed with ChCl in a 2:1 ratio), stabilized by sorbitan monooleate (Span60). The different DES systems possess widely different viscosities (from 37 cP for EG:ChCl to 750 cP for urea:ChCl), enabling the study of how the dispersed phase viscosity influenced the resulting emulsion and monolith structure. The highest viscosity DES (urea:ChCl) resulted in the most stable HIPE prior to polymerization, while also yielding the monolith with optimal mechanical properties; other DES systems gave fragile structures that readily crumbled. The styrenic polyHIPEs prepared from the urea:ChCl DES had an elastic modulus of 3685 psi and crush strength of 218 psi; the porosity and mechanical properties of the material were readily tuned through varying the surfactant concentration.

**Figure 9:**
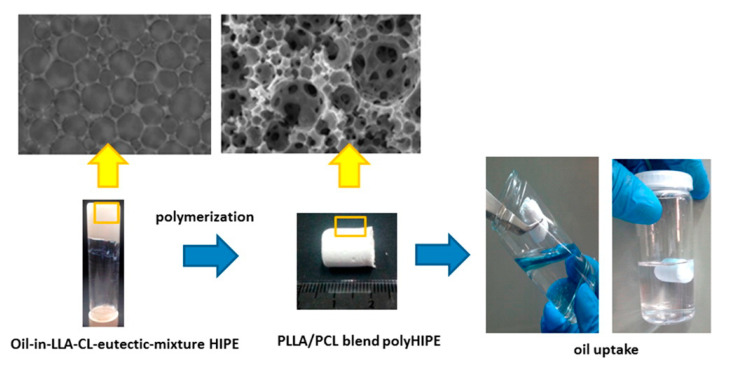
Oil-in-Eutectic polymerized high internal phase emulsions (polyHIPEs) prepared via the emulsification of tetradecane with a 30:70 mol/mol mixture of l-lactide (L-LA) and ε-caprolactone (CL) and subsequent ring opening polymerization of the continuous phase. The resulting polyHIPEs were subsequently used as absorbent materials for hydrocarbon uptake. Reproduced with permission from [83], Copyright 2016, American Chemical Society.

Pérez-Garcia et al. prepared polymerizable eutectics based on l-lactide (L-LA) and ε-caprolactone (CL) in a 30:70 ratio and subsequently used them as the continuous phase for the preparation of HIPEs where tetradecane was the dispersed phase [83] (Figure 9). This represents a contrast to the above work, where the eutectic is typically the dispersed phase. DBU and methanesulfonic acid (MSA) were used as catalysts for the ring opening polymerization of this eutectic at 37 °C with BnOH as initiator. As DBU and MSA are selective catalysts for the homopolymerization of LLA and CL, the resulting polyHIPE was a homopolymer blend as confirmed by NMR and MALDI-TOF mass spectrometry. Due to the polymerization temperature, the resulting PCL was highly crystalline (~90%), whereas the PL-LA was of lower crystallinity (~30%). The polyHIPEs formed had pores of diameter ~20 μm with a degree of openness ~8% and were used as adsorbent materials with respect to the uptake of various hydrocarbons at high capacity (between 2.0 g g^−1^ and 2.5 g g^−1^) and good recyclability.

### 2.4. Molecularly Imprinted Polymers (MIPs)

Several groups have reported the use of DES systems for the design of MIPs and/or nanoparticles that carry MIP functionality [125,126,127,128]. The supramolecular nature of DES systems and strong hydrogen bonding network is particularly appealing for the design of MIPs, given that their preparation relies on specific, selective non-covalent binding of a templating molecule.

MIPs are commonly used to adsorb bioactive or pharmaceutically relevant compounds. For example, Li and Row [125] prepared MIPs from a ternary DES consisting of caffeic acid (CA), formic acid (FA) and ChCl prepared in a wide variety of compositions which were prepared using the antibiotic levofloxacin as a template. The levofloxacin was present in equimolar quantities to the ternary DES. CA was polymerized by the addition of AIBN in the presence of EDMA as cross-linker and porogenic solvent (ethanol:water 9:1). The polymerization yielded spherical polymer particles of the order of 300 nm diameter with specific surface areas ranging from ~6 m^2^ g^−1^ to 11 m^2^ g^−1^ and pore sizes from 118 Å to 308 Å. The MIPs showed high adsorption capacities for levofloxacin present in aqueous solution and millet extracts (>5000 ug g^−1^). The same DES was also used to successfully prepare MIPs with high recognition capability for the polyphenols protocatechuic acid, catechin and epicatechin [129].

Li et al. [128] used a 2:1 glycerol:ChCl DES to prepare acrylamide-based MIPs via free-radical cross-linking polymerization with EDMA, specifically to template chlorogenic acid, the bioactive compound from Honeysuckle which is commonly found in traditional Chinese medicine. The equivalent polymers were prepared without a templating agent and also via the use of traditional solvents such as methanol and water. Notably, the MIPs prepared in the DES had significantly higher adsorption for chlorogenic acid than those prepared in traditional solvent systems (~13 mg g^−1^ vs. ~10 mg g^−1^, respectively), demonstrating the potential for this synthetic approach.

MIPs are also routinely prepared on nanoparticle supports, in particular magnetic nanoparticles, to facilitate isolation through application of an external magnetic field. This concept has also been used in conjunction with DES systems. Li and Row prepared [130] magnetic nanoparticles with surface-grafted MIPs with two templates for the recognition of commercially relevant polysaccharides (fucoidan and alginic acid) from seaweed in seven different DES systems. The MIPs grafted from the surface of iron oxide nanoparticles in this work were prepared by the cross-linking polymerization of methacrylic acid (MAA) and EDMA. Similar to previous work of the authors, the use of DES systems resulted in MIPs with greater extraction efficiency than the equivalent polymer synthesis in conventional solvents or in bulk, with the extraction capability of the order of ~20 ug g^−1^ for both polysaccharides. Row’s group has also used a similar approach to extract and separate various catechins from black tea ((+)-catechin, (−)-epicatechin, and (−)-epigallocatechin gallate) using magnetic nanoparticles functionalized via the cross-linking polymerization in either MAA:ChCl or MAA:betaine:water DES systems [127]. The protein bovine haemoglobin (BHb) has also been used as a template for preparation of magnetic nanoparticles with surface-grafted MIPs through polymerization in a MAA:ChCl DES [131]; the adsorption capability of these particles for selective recognition of BHb was up to a factor of four greater than those prepared in the absence of a templating agent. The polymerization of vinyl pyrrolidone (VP) and malonic acid in various ChCl-based DES systems in the presence of l-asparaginase (L-ASP) as a templating agent has also been performed [132] to prepare biocompatible MIPs based on magnetic separation.

## 3. Conclusions and Outlook

From their reported discovery close to two decades ago, DES systems have emerged as an exciting alternative to traditional solvents in numerous aspects of chemistry. The fundamental studies regarding the design of these solvents and their subsequent properties have paved the way for their use in specific applications in several areas. What makes their application different in polymer science is their ability to not just serve as a solvent, but also as a reactant and templating agent, which greatly expands their utility. This “polymerizable eutectic” concept has been heavily explored in various settings in material synthesis.

There is no doubt regarding the appeal in using DES systems in place of traditional solvents—they address numerous aspects of green, sustainable polymer and material synthesis, while also providing positive benefits in areas such as thermal and mechanical properties of the resulting polymer, as well as improved reaction kinetics. This combination should see an increase in their use in polymer synthesis in the coming years. Indeed, there are exciting opportunities for their use that have yet to be explored. In our opinion, photopolymerization in DES systems is particularly exciting, given the relatively benign conditions that can be used for photopolymerization, in addition to providing spatial and temporal control of the reaction. Photomediated RDRP techniques [133,134,135] have not yet been explored in DES systems, most likely due to the relatively small number of reports regarding RDRP of any kind. Greater understanding of photopolymerization in DES systems, coupled with their high viscosity, makes them suitable for stereolithography and so is a potential area of future research.

In addition to their appealing physical and chemical properties, DES systems can be prepared from inexpensive natural, renewable or bio-sourced starting materials. To address the desired push to move away from organic solvents derived from petroleum fractions, this is incredibly important. Sustainability in materials synthesis, however, is only partly addressed if the solvent addresses the principles of green chemistry, but the monomer(s) do not. To that end, we believe that the polymerization of bio-derived monomers (such as important commodity monomers such as acrylates, methacrylates and styrenics) in DES systems is an important step forward in truly green polymerization processes. Numerous monomer classes [136], including high and low glass transition temperature [137,138,139] and low glass transition temperature acrylates and methacrylates for industrial applications, have been prepared using lignin derived from hardwood and lignocellulosic biomass; however, the reaction conditions for the subsequent polymer synthesis remain harsh—high temperatures, reflux conditions and the use of volatile organic solvents (VOCs) and halogenated solvents. There is, therefore, an obvious opportunity to explore bio-based polymer synthesis in environmentally friendly, renewable solvents.

Lastly, polymer composites prepared in DES systems will be an active area of research moving forward, given the ability to incorporate composites such as carbon nanotubes and lignin at high concentrations in these systems. Polymer composites are highly industrially relevant given the need to design materials with tailored mechanical, thermal and/or optical properties that surpass those of neat polymers or polymer blends [140]. The dispersion of common nanoparticles such as silica, titania, in addition to various clays and 2D carbon materials such as graphene and graphene oxide is an exciting area of future research, in particular their dispersion into polymerizable eutectics that can enable in situ composite formation to occur. Such application-driven research will no doubt be complemented by fundamental studies on polymerization mechanism in DES systems to achieve exciting breakthroughs in polymer synthesis in the coming years.

## Data Availability

Not applicable.
